# Universal Flu mRNA Vaccine: Promises, Prospects, and Problems

**DOI:** 10.3390/vaccines10050709

**Published:** 2022-04-30

**Authors:** Andrei A. Deviatkin, Ruslan A. Simonov, Kseniya A. Trutneva, Anna A. Maznina, Elena M. Khavina, Pavel Y. Volchkov

**Affiliations:** 1 The National Medical Research Center for Endocrinology, 117036 Moscow, Russia; andreideviatkin@gmail.com (A.A.D.); trutneva-k@mail.ru (K.A.T.); 2 Genome Engineering Lab, Life Sciences Research Center, Moscow Institute of Physics and Technology (National Research University), 141700 Dolgoprudniy, Russia; simonov.ra@phystech.edu (R.A.S.); aamaznina@gmail.com (A.A.M.); khavina.lx@phystech.edu (E.M.K.)

**Keywords:** mRNA vaccine, universal flu vaccine, circRNA, influenza virus

## Abstract

The seasonal flu vaccine is, essentially, the only known way to prevent influenza epidemics. However, this approach has limited efficacy due to the high diversity of influenza viruses. Several techniques could potentially overcome this obstacle. A recent first-in-human study of a chimeric hemagglutinin-based universal influenza virus vaccine demonstrated promising results. The coronavirus pandemic triggered the development of fundamentally new vaccine platforms that have demonstrated their effectiveness in humans. Currently, there are around a dozen messenger RNA and self-amplifying RNA flu vaccines in clinical or preclinical trials. However, the applicability of novel approaches for a universal influenza vaccine creation remains unclear. The current review aims to cover the current state of this problem and to suggest future directions for RNA-based flu vaccine development.

## 1. Introduction

### 1.1. Influenza A Virus Diversity

**Figure 1:**
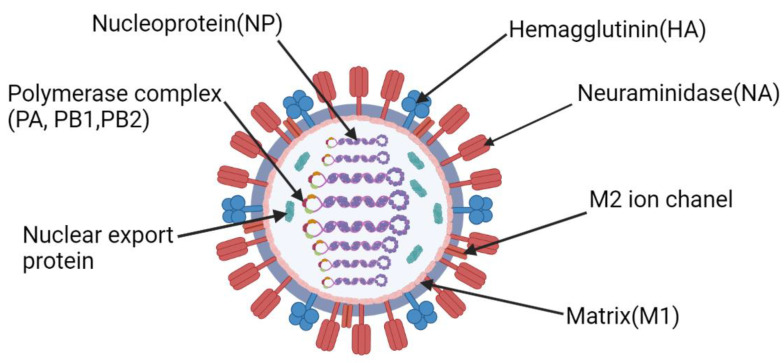
Structure of the influenza virus. Figure was created using BioRender tool.

The influenza virus (Figure 1) is a highly heterogeneous group of negative-sense ssRNA viruses of the Orthomyxoviridae family. To date, four species of influenza viruses, influenza A, B, C, and D 1 (IAVs, IBVs, ICVs, IDVs, respectively), are recognized by the International Committee on Taxonomy of Viruses (ICTV, 10th report). Such division is based on the antigenic properties of their matrix (M) and nucleoprotein (NP) [1,2]. 

All previous human influenza pandemics have been caused by IAV [3]. Thus, the influenza A virus poses the most burden to humankind across influenza viruses [4]. This is primarily caused by the vast diversity of the potential wildlife reservoirs of IAVs [2]. Host shifting inevitably leads to the emergence of novel viral variants with different phenotypic properties. IAVs have been found in many animal species such as IBVs in seals and horses and ICVs and IDVs in pigs. Traditionally, birds are the primary reservoir of IAVs. Indeed, 16 out of 18 hemagglutinin (HA) subtypes circulate mainly in birds [5], whereas 2 out of 18 HA subtypes circulate in bats [6].

**Figure 2:**
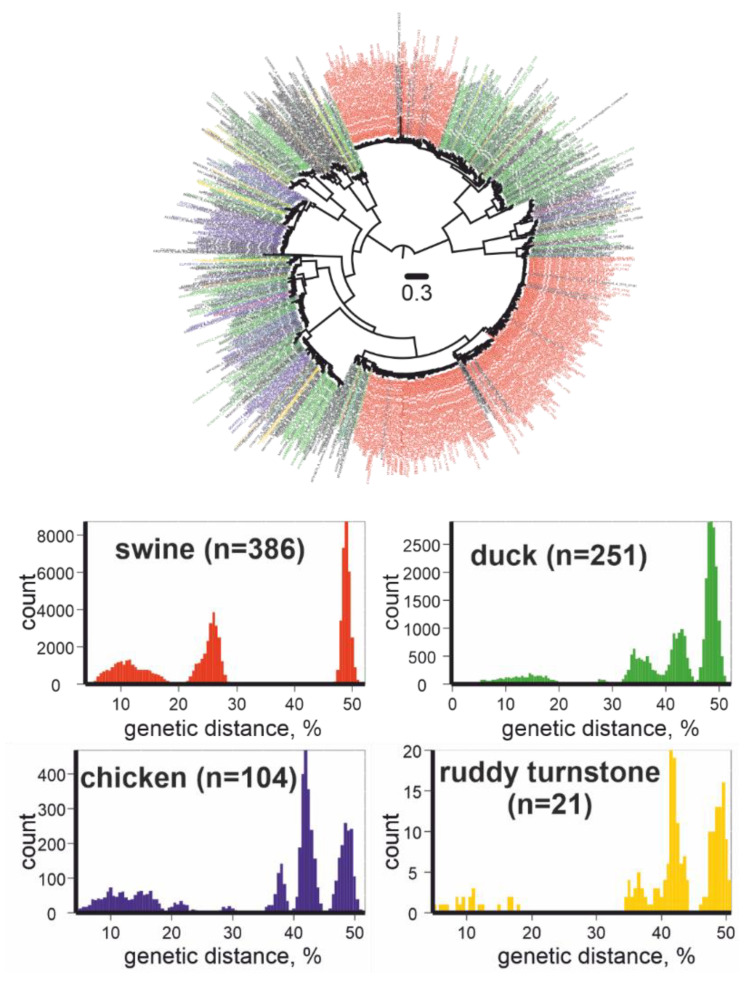
Upper panel: Unrooted maximum likelihood tree for IAVs (HA gene fragment, *n* = 1003). Red color indicates viruses collected from swine, green from ducks, blue from chickens, yellow from ruddy turnstones. Phylogenetic inference was performed using IQ-TREE [7]. Lower panel: pairwise genetic distances for IAVs collected from swine, ducks, chickens, and ruddy turnstones.

The high mobility of birds leads to a global spread and further diversification of IAVs. As a result, the genetic diversity of IAVs is exceptionally high; however, knowledge of natural IAV diversity is rather fragmented [5]. To demonstrate this, we downloaded all available GenBank complete HA sequences of IAVs (*n* = 15,772,) as of September 14, 2021, and omitted sequences similar to each other, at more than 95%, and illustrated their phylogenetic relationships (Figure 2, upper panel). The four most represented host animals were pigs (386 out of 1003 sequences), ducks (251 out of 1003 sequences), chickens (104 out of 1003 sequences), and ruddy turnstones (21 out of 1003 sequences). The 383 sequences isolated from pigs were grouped into two subtypes (H1, H3), and three sequences of viruses collected from pigs were representatives of three other subtypes (H5, H7, H10). A total of 21 sequences from ruddy turnstones belonged to eight subtype (H2, H3, H6, H7, H9, H11, H12, and H13) clusters. Moreover, the genetic divergence was similar in all four groups of viruses, despite the disparate scales of virus detection in farm and wild animals (Figure 2, lower panel). This may indicate that the diversity of IAVs is known to some extent in “human companion” animals and is barely represented in wild animals.

### 1.2. Influenza Transmission from Animals to Humans

Influenza is a classic example of a zoonotic disease that can infect humans directly from avian reservoirs or through other mammals [8,9]. In the case of acquired IAV transmissibility between people, humanity faces novel influenza epidemics and pandemics.

The phenotypic diversity of influenza viruses primarily emerges from two mechanisms: antigenic drift or accumulation of substitutions in surface proteins (HA and neuraminidase, NA) and antigenic shift or segment shuffling (reassortment) [10]. Reassortment of viruses with known phenotypic features may generate novel IAV variants with unpredictable properties. For example, H1N1pdm2009, which caused the last swine flu pandemic, emerged due to reassortment between swine, human, and avian viruses in pigs as hosts [11].

The spillover of a virus from animals to humans is a random event. In the case of influenza, various subtypes of IAVs may infect humans. There are several approaches to predict the next subtype of influenza with pandemic potential [12,13,14,15]. Based on such a prognosis, the manufacturing of appropriate vaccines is initiated that lasts several months. It is noteworthy that modern seasonal influenza vaccines are efficient against antigenically related IAVs. However, predictions on the coming variant of the seasonal flu are not always correct. For example, in 2014, the influenza A (H3N2) component of the 2014–15 Northern Hemisphere seasonal vaccine significantly differed from most circulating IAVs (primarily other H3N2 variants); the efficiency of globally used vaccines was 19% [16].

### 1.3. Advantages and Limitations of Classical Influenza Vaccines

Different types of influenza vaccines, including inactivated whole virus vaccines, live attenuated virus vaccines, virosome vaccines, split-virion vaccines, and subunit vaccines have been in use for many years, exposing both advantages and limitations of the techniques [17]. 

Inactivated flu vaccines have been used for morbidity prevention since the 1940s. According to this method, the pathogen is cultured in an appropriate substrate and then its disease-producing capacity is destroyed by chemical or physical treatment. Such vaccines contain antigens of a live natural virus, thereby forming the most complete repertoire of antibodies, while being unable to infect cells and multiply. At the same time, there are several types of inactivated vaccines that have proven their efficiency: whole particle virus [18], split virus [19,20], and subunit [21] vaccines. 

Live attenuated influenza vaccines (LAIV) primarily include vaccines against influenza A(H1N1), subtype A(H3N2), and the Victoria strain of influenza B. LAIV are obtained by repeatedly passaging the parental influenza virus into virus-susceptible cells or embryonic chicken eggs. Such an approach has been used since the 1950s [22]. As a result, less virulent strains of the influenza virus are obtained due to adaptation to conditions not present in the human organism. This type of influenza vaccine strain has a low virulence and pathogenicity but still allows the virus to infect cells and multiply within a relatively short period of time after vaccination [23]. It is important to consider that live vaccines have an unpredictable risk of reversion into a pathogenic virus due to random mutations [24] and recombination or reassortment with wild type viruses [25]. With the development of reverse genetics techniques, it has become possible to design viruses that carry several desired foreign genes [26]. Using these modern methods, trivalent and quadrivalent LAIVs can be designed to express three or four influenza virus HA genes, respectively.

Recently, recombinant HA vaccines were approved for influenza prevention [27]. This type of vaccine is manufactured by HA protein expression in the baculovirus–insect cell system. Accumulated data suggest that this approach is more effective than inactivated vaccines. For example, Richards et al., demonstrated that recombinant HA-based vaccine induced enhanced CD4 T cell responses and HA-specific antibodies compared with split and subunit vaccines [28]. 

Currently, there are no other platforms approved for human influenza virus vaccine implementation. However, existing approaches have not solved the obstacle of the low influenza vaccination effectiveness. A potential solution to this problem is the development of a universal influenza vaccine that would provide effective immunity against all strains of the virus.

There are other promising approaches for developing an influenza vaccine. The first commercial DNA vaccine against H5N1 in chickens was approved by the United States Department of Agriculture in 2017 [29]. However, DNA vaccines have a number of inevitable disadvantages, including unmethylated CpG-motifs serving as a signal for foreign DNA elimination by the immune system in vivo [30]. After entering the cell, foreign DNA must somehow enter the nucleus through an additional barrier, the nuclear membrane, to produce mRNA that should be exported from the nucleus [31]. 

**Figure 3:**
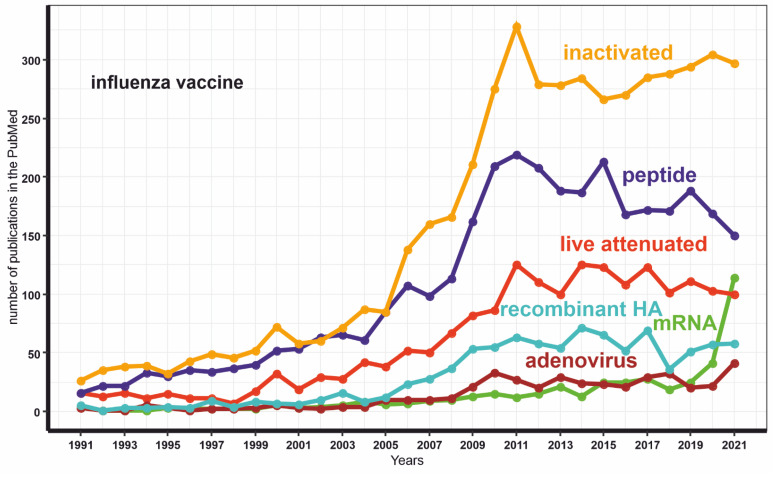
The dynamics of the publications number with keywords “mRNA INFLUENZA VACCINE” or “live attenuated INFLUENZA VACCINE” or “inactivated INFLUENZA VACCINE” or “adenovirus INFLUENZA VACCINE” or “peptide INFLUENZA VACCINE” or “recombinant HA INFLUENZA VACCINE”.

Conversely, mRNA-assisted delivery does not have such drawbacks. Notably, the number of publications dedicated to mRNA-based platforms extensively increased after the successful implementation of several mRNA vaccines against COVID-19 (Figure 3). Herein, we have reviewed whether this method has prospects for creating a universal flu vaccine.

## 2. mRNA Vaccine

### 2.1. Development and Use of mRNA Vaccines

Thirty-five years ago, the possibility of protein expression in human cell lines from exogenous mRNA mixed with fat droplets was demonstrated [32]. A year later, the possibility of mRNA delivery into a living organism (frog embryo) was revealed. However, for many years, mRNA was not considered as a viable method to prevent or treat disease due to the perceived technical sophistication. Despite the lack of visible applications of the new technology, several scientific groups continued to consider using mRNA as a delivery vehicle, e.g., for cancer vaccines [33]. 

An unexpected impetus to the development of mRNA vaccines was provided by the COVID-19 pandemic. There were no ready-made solutions at the time of the emergence of a demand for a vaccine against a novel infection. In such a situation, standard time-tested approaches require comparable, if not more, time in development as compared with new ones. Currently, two major mRNA vaccines in use are manufactured by Moderna and Pfizer [34]. Both vaccines are based on a similar principle. The Moderna and Pfizer vaccines were administered to hundreds of millions of people, efficiently protecting against moderate and symptomatic SARS-CoV-2 infection [35]. It should be noted that over time the effectiveness of vaccination inevitably decreases [36], which necessitates the use of revaccination.

### 2.2. mRNA Vaccine Method of Action and Delivery

#### 2.2.1. Types of mRNA Vaccines

**Figure 4:**
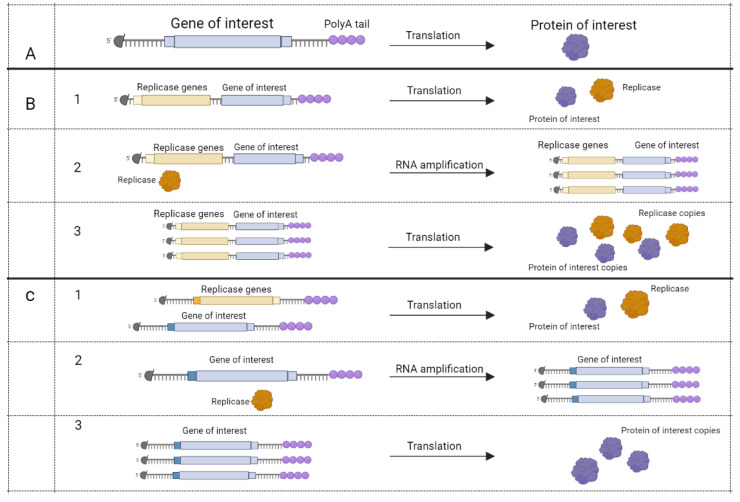
mRNA vaccines’ method of action [40]. Five-prime cap (black circle) and polyA tail present on all mRNAs. (**A**), conventional mRNA encodes protein that may be used as an antigen.Apart from the gene of interest, self-amplifying mRNA (SAM) may encode RNA dependent RNA polymerase (RDRP) at the same molecule (**B**) or at the other mRNA (**C**). (**B**,**C**), step 1, translation of RDRP and gene of interest. Step 2, RDRP amplifies mRNA. Step 3, translation of self-amplified mRNA. Figure was created using BioRender tool.

Based on their method of action, mRNA vaccines can be divided into two types: conventional mRNA and self-amplifying mRNA (SAM) vaccines [37]. Conventional mRNA vaccines take advantage of cellular machinery to translate the appropriate protein, whereas SAM vaccines have coding accessory proteins for self-replication (RNA-dependent RNA polymerase, capping enzymes, proteases) beside target RNA [38,39,40,41] (Figure 4). 

SAM vaccines may be used in the form of formulated in vitro transcribed mRNA or in a form of viral replicon particles produced by packaging SAM with alphavirus structure proteins in cell culture [42]. This was demonstrated through the development of vaccines that prevent CMV infection [43]. Notably, the presence of alphavirus proteins may be toxic for the host cells [44]. At the same time, a recent safety study of the SAM rabies vaccine in rats showed that SAM was well tolerated by the animals [45].

Hekele and colleagues have proven the safety, tolerance, and efficacy of a SAM vaccine coding for influenza hemagglutinin H1N1. This vaccine protected mice from inoculation by the H7N9 strain. Such preparations may be generated within 8 days in a cell-free way after the discovery of a new viral strain [46]. Another advantage of such a platform is the ability to use low concentrations of nucleic acids. Moreover, the amplification of delivered mRNA occurs in situ, generating stronger protective immunity to the antigen as compared with conventional mRNA vaccines. 

In addition, the new cationic nanoemulsion (CNE)-formulated SAM vaccine against another RNA-virus, the Zika virus, has recently been shown to protect a non-human primate model in a preclinical trial [47]. Several studies demonstrated the great potential of SAM vaccine usage for preventing COVID-19. Strong stimulation of either humoral or cellular immune response was revealed [48,49]. Notably, one SAM vaccine is undergoing Stage I clinical trials after confirmation of effectiveness against different SARS-CoV-2 strains [48]. Thus, it can be said that SAM vaccines have shown their immunogenic potency against multiple targets [46,47,48,49,50,51,52]. In addition, based on preclinical data, it can be concluded that SAM-mRNA vaccines could potentially induce a more durable immune response compared to non-replicating mRNA vaccines [53].

**Figure 5:**
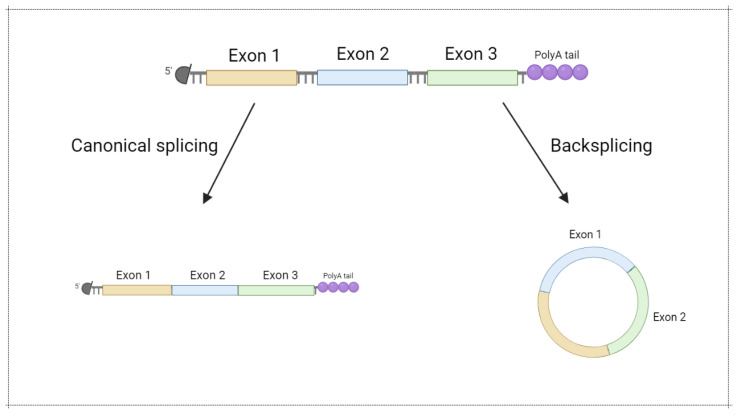
Circular mRNA generation via backsplicing. Modified from [59]. Figure was created using BioRender tool.

**Figure 6:**
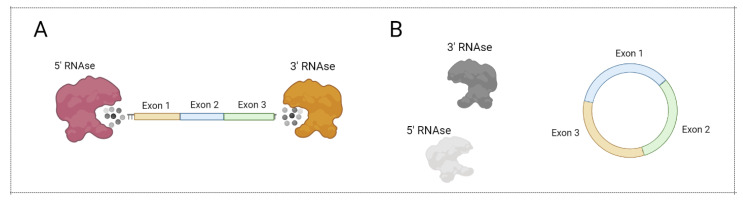
CircRNA protection from exonuclease action. (**A**) Linear mRNA can be degraded by 5’ RNAse and 3’ RNAse. (**B**) Circular mRNA cannot be degraded by 5’ RNAse and 3’ RNAse. Figure was created using BioRender tool.

Most ribonucleases involved in mRNA degradation have 5’- or 3’-end-dependent activity. As a result, the stability of linear mRNA is low, impairing mRNA-based vaccine implementation. Notably, circular forms of RNA (circRNA) were demonstrated to be an abundant and prominent type of RNA generated in the result of backsplicing (Figure 5) [54]. Due to the lack of a 5’- and 3’-end, circRNA are more stable in comparison with linear mRNA (Figure 6). Recently, several approaches for circRNA generation have been suggested [55,56,57,58]. 

RNA circularization leads to enhanced stability of the mRNA and greater efficiency in the production of the target protein [55]. Moreover, a circular mRNA vaccine could be stored at room temperature [60]. As a result, manufacturing and implementation costs would be significantly lower. To the best of our knowledge, there are currently no data about circular mRNA vaccines published in peer-reviewed journals. However, some commercial companies are raising funds to implement the concept of protein translation from circular RNA in humans. The Laronde startup raised hundreds of millions of dollars in 2021 for the development of “endless RNA therapeutics” [61]. 

#### 2.2.2. Peculiarities of mRNA Vaccine Synthesis

In essence, mRNA vaccine production is an attempt to simulate the changes that mRNA undergoes in a human cell, using a cascade of biochemical reactions in a cell-free environment. For example, mRNA-1273 (Moderna) is generated by an in vitro T7 RNA polymerase-mediated transcription process from plasmid DNA [62]. The DNA template contains a codon-optimized immunogen coding sequence, 5′ untranslated region (UTR), 3′ UTR sequences, and a polyA tail. It should be noted that the miRNA binding sites in the 5’ and 3’ UTRs decrease the half-life of mRNA as well as vaccine antigen expression in target cells and tissues. Additionally, any non-canonical start codons or strong secondary structures interfering with translation initiation at the 5’ UTRs may lower the translation efficiency of a specific antigen. To enhance expression and avoid innate immune hyperactivation, ORF should also be optimized with codon usage and GC content.

In addition, uridine (U) 5′-triphosphate is 100% substituted by 1-methylpseudouridine (m1Ψ) 5′-triphosphate in the transcription reaction mixture [63]. As a result, all Us in the sequence of mRNA-1273 are changed to m1Ψs. It should be noted that U-to-m1Ψ substitution has no effect on the translated protein sequence. Such modification was demonstrated to significantly enhance the mRNA translation level [64]. However, inclusion of m1Ψ guarantees less activation of the innate immune system, compared to mRNA containing canonical uridine [63]. Indeed, in vitro transcribed RNA induces innate immune response through the interaction of cytosolic or endosomal RNA-sensing pattern-recognition receptors. These proteins interact with single- or double-stranded RNA containing exogenous pathogen-associated molecular patterns. Such interactions drive expression of pro-inflammatory cytokines, chemokines, type I IFN, and interferon-stimulated genes [65,66]. Nevertheless, inhibition of early I type IFN-derived immune response is critical for enhancing immunization efficiency by an mRNA vaccine [67]. At the same time, hyperstimulation of the innate response can lead to the elimination of exogenous mRNA or the blockage of its translation mechanism [68]. Therefore, mRNA vaccines must be designed in such a way as to "moderately" stimulate the immune response. Chemical modifications of nucleosides naturally occurring as post-translational modifications in cells may allow the prevention of Toll-like receptor-mediated activation of the immune response [69]. Thus, the use of a novel modification assists in the avoidance of excessive activation of the innate immune response. An example of the potential importance of including a modification in mRNA is the COVID-19 mRNA vaccine produced by CureVac. This vaccine demonstrated low effectiveness (around 48%) [70]. The low efficacy of the drug was likely due to the lack of m1Ψ modification as compared with the Moderna and Pfizer vaccines [71]. 

The RNA resulting from in vitro transcription undergoes a biochemical 5’ capping procedure, imitating the natural process that occurs in the nucleus of the cell [72]. Artificial capping could be conducted by using commercially available kits, e.g. CleanCap (Trilink Biotechnologies) [73]. While such a modification stabilizes the synthesized RNA by shielding from 5’ RNA exonucleases, it is necessary for cap-dependent translation initiation. Circular mRNA vaccines that have yet to come into practice are, by definition, incapable of cap-dependent translation initiation. However, there are alternative cap-independent mechanisms of translation initiation, mediated by internal ribosomal entry sites (IRES) or N6-methyladenosine (m6A) modification incorporated into the 5’ UTR region of mRNA [74,75]. 

T7 RNA polymerase, in addition to its primary function, is capable of synthesizing RNA based on an RNA template (i.e. RNA template-directed RNA synthesis) in significant quantities [76]. As a result, an RNA strand complementary to the template is synthesized, generating dsRNA. The entry of double-stranded RNA into a cell inevitably leads to a strong immune response, initiated by the binding of a foreign molecule to receptors, such as RIG-I-like receptors [77]. As a result, the efficacy of translation is seriously reduced [78]. The additional step of removing the dsRNA fraction circumvents this problem. Thus, U-to-m1Ψ substitution and dsRNA removal are two necessary and sufficient steps to regulate the interaction between vaccine mRNA and immunity [78]. At the same time, it should be noted that the difficulty of scaling up mRNA vaccine production is of particular concern [79]. This problem remains the subject of active research [80,81].

#### 2.2.3. Delivery Vehicles 

Various mRNA delivery platforms may be used, depending on the task. Viral vectors, gene guns, electroporation, penetrating peptides, polymers, and liposomes are among the most widespread approaches [82,83]. Naked unformulated mRNA was shown to be expressed in cells [84], however cellular uptake is less than 0.01% [85]. Notably, most of these methods are only suitable for in vitro purposes.

Liposomes are the first delivery approach used in nanopharmaceuticals as a kind of universal carrier for both hydrophobic and hydrophilic cargoes: proteins, nucleic acids, and small molecules [86]. A liposome is a 20–1000 nm formation with one or more lipid bilayer containing aqueous and hydrophobic compartments. In 1994, Harashima et al., demonstrated a positive correlation between the size of such particles and the opsonization and macrophage phagocytosis uptake in vivo [87]. This means that liposome size should be carefully measured for delivering purposes [88].

To date, the most common way of delivering mRNA for therapeutic purposes was lipid nanoparticle (LNP) injection. LNP consists of positively charged cationic and ionizable lipids that mediates complexing with negatively charged mRNA. Moreover, such formulation is necessary for cellular uptake and endosomal escape [89,90]. Cationic lipids in LNPs are neutralized with anionic cell lipids and promote nucleic acid entering the cytoplasm through the disruption of LNP complexes. Although LNPs are non-immunogenic and non-toxic in comparison to viral vectors, they still have some cytotoxicity [91] that is dependent on the structure of hydrophilic heads or PEG (polyethylene glycol) modifications of lipids, which are widely used to increase in vivo stability [92]. This may cause membrane damage or vacuolization of the cytoplasm and may affect important cellular pathways and cell cycle stages. There are many new lipids under development for lowering cytotoxic effects without decreasing delivering efficiency.

Producing LNPs is difficult and hardly scalable. LNPs themselves are not sufficiently stable, sterilizable, or bioavailable [86]. Solid lipid nanoparticles (SLNs) containing solid lipids instead of liquid crystalline and nanostructured lipid carriers (NLCs) containing a mix of solid and liquid crystalline lipids were developed for overcoming those limitations.

Another non-viral delivery platform, cationic nanoemulsion (CNE), consists of cationic lipid DOTAP (1,2-dioleoyl-sn-glycero-3-phosphocholine), emulsified with components of Novartis’s proprietary adjuvant MF59 and is well tolerated in every age group [50]. CNE’s advantages in delivering and enhancing vaccine potency and safety have been proven in over 100 clinical trials. Additionally, it can be stored at +4 °C for up to 3 years [47].

It should be noted that there is a possibility for targeted LNP delivery with the use of incorporated ligands for cell receptors (natural ligands, antibodies, aptamers) and stimuli-responsive (pH, temperature, magnetic fields, laser irradiation) LNPs, as it has been tested for anti-cancer therapies [86]. 

**Figure 7:**
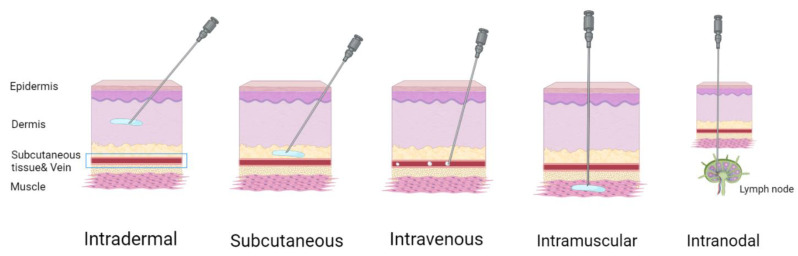
Administration routes of mRNA vaccines. Figure was created using BioRender tool.

There are different administration routes (Figure 7) for mRNA-based therapeutics [90]. Systemic intravenous administration often leads to the accumulation of LNPs in the liver. This is acceptable for replacement therapies and for the production of specific anti-pathogen or anti-cancer antibodies due to inherent liver capability for protein secretion. Otherwise, intravenous delivery may cause a wide distribution of nanoparticles through the lymph nodes, enhancing immune response to the antigen, compared with local administration; this could cause adverse effects, therefore using targeted nanoparticles is preferable. Direct local administration (intramuscular, intradermal, subcutaneous) of mRNA-containing nanoparticles into the target tissue is preferential for achieving a local therapeutic effect and a systemic effect through the recruiting of local antigen presenting cells. Local injection is commonly used for vaccination purposes. Van Lint et al., have shown that direct intranodal administration of tumor-associated antigen mRNA together with mRNA coding for immunomodulatory proteins causes a robust T cell response mediated by dendritic cell mRNA uptake, translation, and antigen presentation [93]. At the same time, intranodal delivery of mRNA-encoded influenza nucleoprotein activates an effective cross-strain T cell response in mice [94].

Some LNP-based therapeutics are already clinically approved and there are also several nucleic acid-based approaches in use. For example, Onpattro by Alnylam Pharmaceuticals, which is a transthyretin-directed siRNA formulated with LNP for the treatment of polyneuropathy caused by hereditary transthyretin-mediated amyloidosis (hATTR amyloidosis), and, of course, the commonly known anti-COVID-19 mRNA vaccines, BNT162b2 by Pfizer/BioNTech and mRNA-1273 by Moderna, although they only received Emergency Use Authorization (EUA) in 2020 [86]. There are also other Stage I and II clinical trials of mRNA-based LNP therapeutics against tumors (melanoma, breast cancer, ovarian cancer, glioblastoma, solid tumors, etc.), viral infections (rabies, Zika virus, CMV, Influenza virus, SARS-CoV-2), tuberculosis, and others [86].

#### 2.2.4. Flu mRNA Vaccines under Development

There are four influenza mRNA candidate vaccines in clinical trials proposed by Sanofi/TranslateBio, Pfizer, Moderna, and NIAID. The Sanofi/TranslateBio vaccine is a monovalent vaccine that codes for the hemagglutinin protein of A/H3N2. Pfizer’s medical has two monovalent vaccines that code for the hemagglutinin of H1N1 and B/Yamagata lineage AIV combined into a singular bivalent vaccine.

Moderna’s candidate, mRNA-1010, is a quadrivalent vaccine, encoding the hemagglutinin for two IAVs and two IBVs, selected based on WHO recommendation: A/H1N1, A/H3N2, B/Yamagata-, and B/Victoria-lineages [95]. NIAID’s candidate, FluMos-v1, is a universal mRNA vaccine that stimulates antibodies against several different strains of the influenza virus through the display of a hemagglutinin fragment on the surface of a self-assembling nanoparticle scaffold [96]. 

In addition to the above examples, several other prototypes are being developed [97]; thus, there are at least 10 mRNA vaccines for the influenza virus currently in pre-clinical trials, including a multivalent Moderna vaccine. 

In other words, the possibility of using mRNA vaccines is already the subject of active study and several prototypes are being tested in humans. It should be noted that mRNA vaccines for the influenza virus may be a larger challenge to market to the public than for COVID-19 as there are non-mRNA options available in use. However, while current vaccines are safe, their efficacy and coverage leaves space for improvement, which, in theory, can be improved upon by using mRNA vaccines [97]. 

## 3. Universal Flu Vaccine Perspectives 

The influenza virus causes yearly outbreaks, often epidemics, and occasionally pandemics. This occurs due to the high variability of the virus; the type A virus is subject to both antigenic shift and antigenic drift. In this regard, the development of vaccines against individual strains is untenable and there is a need to create “universal” vaccines. According to the National Institute of Allergy and Infectious Diseases (NIAID) the criteria for a universal vaccine are: (1) at least 75% efficacy against symptomatic influenza virus infection; (2) protection against Group I and Group II influenza A viruses (influenza B virus would be a secondary target); (3) durable protection that lasts at least 1 year and preferably through multiple seasons; (4) suitable for all age groups [98]. However, the definition of a universal flu vaccine may vary slightly according to the source [99]. Potentially, universal vaccines can be created in different ways, namely by rational design of new immunogens that guarantee broad protection or by the introduction of vaccines based on a large number of different antigens. Notably, it is currently difficult to speculate on a gold standard platform for universal influenza vaccines due to the diversity of the platforms that have been used for vaccine creation.

Recently, Nachbagauer et al., [100] demonstrated the safety and immunogenicity of LAIV based on chimeric hemagglutinin-based (cHA) techniques in 18-39 year old adults. This study included three experimental groups of patients. Each participant received two doses of the vaccine 85 days apart. Patients from two groups received LAIV expressing a cH8/1 HA and an N1 NA as a first dose and an inactivated influenza virus (IIV) cH5/1 HA with or without adjuvant as a second dose. Chimeric HA consisted of an H1 or H5 stalk domain and an H8 head domain. Patients from the third group received a IIV vaccine expressing a cH8/1 with adjuvant as a first dose and a IIV vaccine expressing a cH5/1 with adjuvant as a second dose. Sequential vaccination with cHA with different head domains but the same stalk domain caused high anti-stalk antibody titers in all experimental groups. Serum anti-stalk IgG serum antibody levels were found to be long lived, stabilizing at a level significantly above baseline at 6 months after vaccination, remaining constant 18 months after the booster shot. Vaccination with cHA vaccines had acceptable safety in adults, while providing strong broad-spectrum antibodies that were functional and long lasting. This clinical trial focused on proof of concept, and therefore only considered Group 1 IAVs. However, the researchers are currently developing IAV Group 2 and IBV cHAs and hope that constructs can be combined into a trivalent vaccine enabling protection for a significantly wider pool of seasonal influenza viruses.

The delivery of mRNA vaccines via lipid nanoparticle encapsulation induced a wide and strong immune response in a murine model [101]. The preparation was a combination of four conservative antigens (hemagglutinin stalk, neuraminidase, matrix-2 ion channel, and nucleoprotein). The immunity provided by a single immunization with nucleoside-modified mRNA-lipid nanoparticle vaccines protected mice from challenges with a panel of Group 1 influenza viruses, which confirmed the broad protective potential of the vaccine. 

In 2019, Eickhoff et al., provided proof-of-concept for a T cell targeted universal influenza vaccine, composed of highly conserved influenza epitopes, which were immunogenic and protective in mice expressing the appropriate human MHC [102]. The novel vaccine induced immunity against matrix (M1, M2) and nucleoprotein (NP) antigens, which are highly conserved among influenza A strains in contrast with HA and NA proteins that change rapidly due to antigenic drift and antigenic shift. In the other study, a cross-strain T cell response was induced by intranodal injection of mRNA encoding influenza nucleoprotein in mice [94]. For the construction of the vaccine, M1, M2, and NP protein sequences of 53 influenza strains were analyzed. Epitopic 9-mer peptides were chosen according to the Conservatrix algorithm. Potential immunogenicity of the selected sequences was assessed by the EpiMatrix algorithm. The resulting high priority conserved influenza immunogenic consensus sequences were produced as synthetic peptides for immunogenicity evaluations, arranged into a synthetic minigene for DNA vaccine preparation. The novel vaccine was tested in HLA transgenic mice. The vaccines were highly immunogenic; moreover, vaccine induced immunity was protective against challenges with the highly virulent H1N1 PR8 and a less virulent mouse-adapted H3N2, which demonstrates the proof-of-principle that conserved T cell epitopes expressed by different strains of influenza can induce heterotypic influenza immunity.

In 2021, Chivukula et al., demonstrated the applicability of the mRNA therapeutic platform for multivalent influenza vaccine creation [103]. Proof-of-concept experiments were successfully completed in mice and nonhuman primates (NHP). The effect of quadrivalent formulations of co-encapsulated in LNPs H1, N1, H3, and N2 mRNA and bivalent (H1, N1 or H3, N2 mRNA) vaccines in NHP demonstrated no difference to equivalent monovalent (H1, H3, N1, or N2) vaccines according to humoral immune response. Robust neutralizing antibody titer was generated for all delivered antigens. This supports the concept of an mRNA multivalent vaccine as a prospective well-controlled scalable platform for fast pandemic or seasonal viral vaccine production. The findings indicate that co-encapsulated and combined multivalent vaccines of HA/NA mRNA-LNPs could efficiently deliver all four antigens without any sign of immunological interference and all antigens were as immunogenic as in the formulation when these antigens were delivered singularly. 

To summarize, there are many promising approaches to universal influenza vaccine development; several are already in clinical trials [100]. There is hope that the efficiency of at least one of the considered approaches will be definitively confirmed in coming years. Each of the aforementioned methods can be delivered via a recently emerged mRNA vehicle platform, e.g., chimeric HA, predicted epitopic peptides, or a cocktail of various IAVs subtypes genome fragments may be inserted into an mRNA cassette. Moreover, multiple mRNA transcripts can be packaged and delivered as a combination with no differences in the impact on the magnitude of humoral immune response, compared with antigen delivered monovalently [103].

## 4. Future Directions

Recently, the Wellcome Trust foundation established funding to develop the influenza vaccine research and development roadmap [99]. This document pays great attention to the emergent and successful experience of COVID-19 vaccine development [99]. The authors of the roadmap demonstrate great expectations on the usage of several new platforms for the development of seasonal and universal influenza vaccines. Particularly, the mRNA-based platform could be a prospective strategy that shortens the time from identifying candidate influenza strains through the seasonal vaccine development and distribution process. However, in our opinion, and according to proof-of-concept results of Chivukula et al., the mRNA strategy is also promising for broadly protective universal influenza vaccine creation. In addition, as mentioned above, great progress has been made in establishing self-amplifying mRNA.

The widespread use of mRNA vaccines is hampered by the instability of mRNA. A potential solution to this problem is the introduction of covalently closed circular mRNAs as a new platform for the delivery of antigen coding sequences to the human organism, followed by translation in situ. Based on available data, it can be concluded that the storage of circular mRNAs is acceptable at room temperature. However, universal influenza vaccines based on this platform have not been previously developed. Therefore, there are potential pitfalls that are currently invisible and the new platform should perhaps be treated with restrained optimism. It should be noted that, on the premise of mRNAs, approaches based on peptide vaccines and protein vaccines can both be used. In addition, the platform potentially allows for the combination of different ideas of products to prevent influenza. 

There are several promising approaches to developing a universal influenza vaccine. It is likely that, in coming years, at least one of them will come into use and become widespread practice. In our opinion, one of the most optimal ways to deliver a therapeutic drug would be by using “mRNA as a vector”.
